# Broad-Range Antiviral Activity of Hydrogen Sulfide Against Highly Pathogenic RNA Viruses

**DOI:** 10.1038/srep41029

**Published:** 2017-01-20

**Authors:** Nikolay Bazhanov, Olivier Escaffre, Alexander N. Freiberg, Roberto P. Garofalo, Antonella Casola

**Affiliations:** 1 Department of Pediatrics, University of Texas Medical Branch, Galveston, TX, USA; 2 Department of Pathology, University of Texas Medical Branch, Galveston, TX, USA; 3 Microbiology and Immunology, University of Texas Medical Branch, Galveston, TX, USA; 4 Sealy Center for Vaccine Development, University of Texas Medical Branch, Galveston, TX, USA.; 5 Center for Biodefense and Emerging Infectious Diseases, University of Texas Medical Branch, Galveston, TX, USA.

## Abstract

Hydrogen sulfide is an important endogenous mediator that has been the focus of intense investigation in the past few years, leading to the discovery of its role in vasoactive, cytoprotective and anti-inflammatory responses. Recently, we made a critical observation that H_2_S also has a protective role in paramyxovirus infection by modulating inflammatory responses and viral replication. In this study we tested the antiviral and anti-inflammatory activity of the H_2_S slow-releasing donor GYY4137 on enveloped RNA viruses from Ortho-, Filo-, Flavi- and Bunyavirus families, for which there is no FDA-approved vaccine or therapeutic available, with the exception of influenza. We found that GYY4137 significantly reduced replication of all tested viruses. In a model of influenza infection, GYY4137 treatment was associated with decreased expression of viral proteins and mRNA, suggesting inhibition of an early step of replication. The antiviral activity coincided with the decrease of viral-induced pro-inflammatory mediators and viral-induced nuclear translocation of transcription factors from Nuclear Factor (NF)-kB and Interferon Regulatory Factor families. In conclusion, increasing cellular H_2_S is associated with significant antiviral activity against a broad range of emerging enveloped RNA viruses, and should be further explored as potential therapeutic approach in relevant preclinical models of viral infections.

Hydrogen sulfide (H_2_S) is a colorless gas that is both toxic and flammable at high concentrations. Despite its toxicity at high doses, H_2_S has been linked to many important physiological functions as gasotransmitter similar to carbon monoxide and nitrogen oxide^1^,^2^. Importantly, H_2_S plays a significant role in various disease states involving inflammation, fibrosis and vascular responses^3^,^4^,^5^. Non-surprisingly, H_2_S has become a target of investigations in life science and a hopeful therapeutic candidate for some diseases, notably the ones involving inflammatory reactions^6^. Hydrogen sulfide is produced at low concentration in mammalian cells by desulfhydration of cystein that involves the action of cystathionine β-synthase (CBS), cystathionine γ-lyase (CSE) or 3-mercaptopyruvate sulfurtransferase (3-MST) (reviewed in ref. 7). Exogenous delivery of H_2_S is achieved either by using sulfide salts, such as sodium hydrosulfide (NaHS), or using other H_2_S-releasing donors. Inorganic hydrogen sulfide salts are not a preferred source of H_2_S as they release an uncontrolled amount of H_2_S in large quantities in relatively short period of time^6^. On the other hand, naturally occurring and lab-produced H_2_S donors such as garlic extracts or derivatives of phosphorodithioate thioaminoacids exhibit a slow and more controlled H_2_S release that mimic physiological settings^6^. Among the synthetic H_2_S-releasing compounds, GYY4137 has been shown to be more water soluble and to release H_2_S by hydrolysis when in contact with solutions^8^. GYY4137 has been studied extensively *in vitro* and proved to be beneficial in *in vivo* models of inflammatory diseases such as after LPS treatment, reperfusion injury, in circulatory shock and as anticancer therapeutic^9^,^10^,^11^,^12^,^13^.

Using an *in vitro* model of airway epithelial cell infection, we recently found that GYY4137 treatment strongly inhibited replication of paramyxoviruses, single-stranded RNA enveloped viruses, specifically Respiratory Syncytial Virus (RSV), human metapneumovirus (hMPV) and Nipah virus^14^. It was also associated with a reduction of pro-inflammatory mediator production, in a manner independent from inhibition of viral replication^14^. In a mouse model of RSV infection, administration of GYY4137 resulted in a significant reduction of lung viral titers and airway inflammation, and in an improvement of lung function and disease outcome^15^. In this study, we investigated whether H_2_S donor antiviral activity would extend to other RNA enveloped viruses. For this purpose, we used an *in vitro* model of highly pathogenic RNA virus infections, including influenza virus (*Orthomyxoviridae*), Ebola virus (*Filoviridae*), Far-eastern subtype tick-borne flavivirus (*Flaviviridae*), Rift Valley fever virus and Crimean-Congo hemorrhagic fever virus (*Bunyaviridae*). Although FDA-approved antiviral treatments are available for influenza virus, the emergence of transmissible resistant variants and the time-dependent effectiveness represent major challenges of the current drugs^16^. No FDA-approved treatment, other than symptomatic, is available for the other four highly pathogenic viruses, as well for many other emerging RNA viruses. Our results show that GYY4137 treatment significantly reduced replication of all tested viruses, leading to a significant decrease of virus-induced pro-inflammatory mediator release in the context of Ebola and influenza virus infection. The decreased inflammatory mediator secretion was associated with a reduction of viral-induced nuclear translocation of transcription factors belonging to the Nuclear Factor (NF)-κB and Interferon Regulatory Factor (IRF) families, which are key regulators of pro-inflammatory gene expression.

## Results

### GYY4137 interferes with replication of influenza viruses

**Figure 1:**
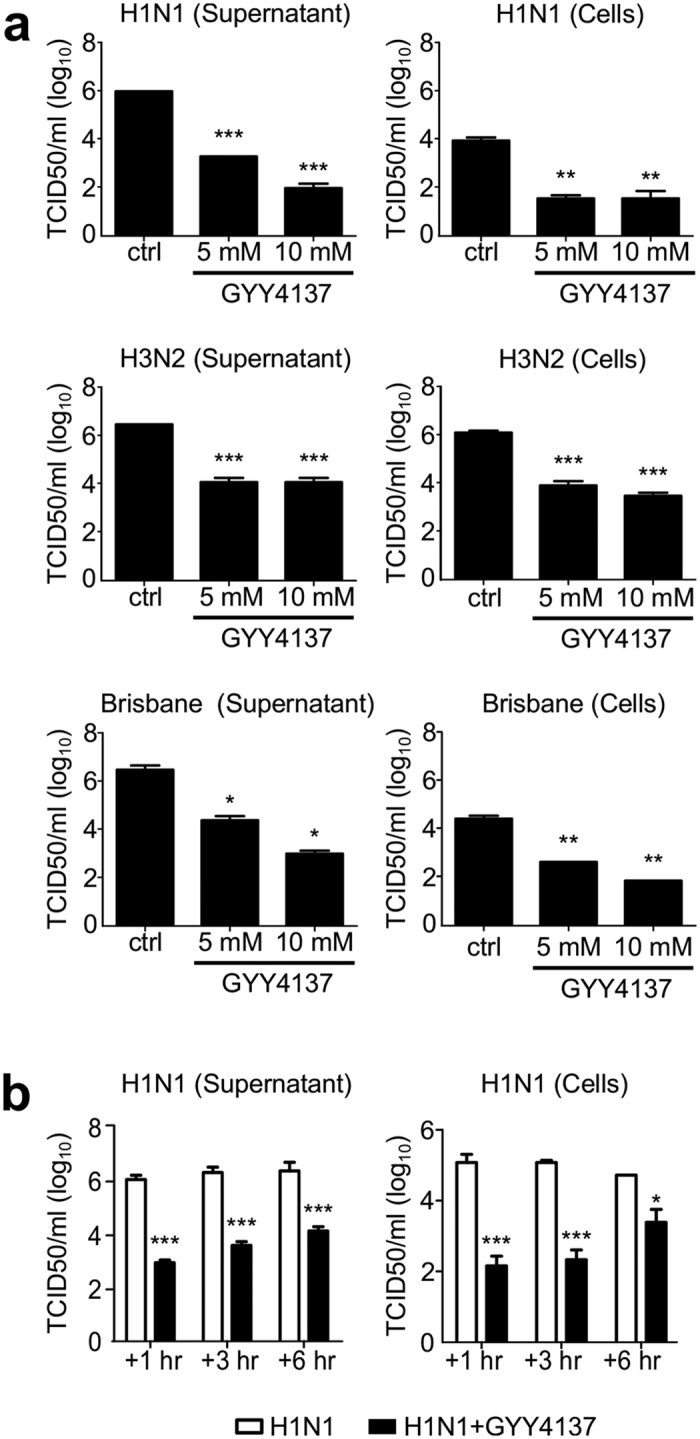
GYY4137 inhibits replication of influenza viruses *in vitro*. (**a**) MDCK cells were infected with influenza A viruses H1N1, H3N2 or influenza B at MOI of 0.01 and treated with 5 or 10 mM of GYY4137 1 h after infection. Cell supernatants and cell pellets were collected at 24 h p.i. to measure viral titers. (**b**) MDCK cells were infected with influenza virus H1N1 at MOI of 0.01 and treated with 10 mM of GYY4137 at 1, 3 or 6 h p.i. Cell supernatants and cell pellets were collected at 24 h p.i. to measure viral titers. The values represent the means of logarithmically-transformed titer values, error bars – standard error of means (SEM), n = 3; *p < 0.05; **p < 0.01; ***p < 0.001 treated versus untreated samples when compared using one-way ANOVA with Tukey post-hoc test.

It is estimated that approximately 28,000 of critical-illness hospitalizations are attributed annually to seasonal influenza^17^. The number of hospitalization increased to approximately 274,000 during 2009 H1N1 pandemic leading to more than 12,000 deaths in the USA only^18^. To investigate the effect of GYY4137 dose on replication of influenza A and B, MDCK cells were infected with H1N1 (A strain), H3N2 (A strain) and Brisbane (B strain) at MOI 0.01 and treated at 1 h post-infection (p.i.) with either 1, 5 or 10 mM of GYY4137. Titers of released and cell-associated viral particles were assessed separately at 24 h p.i. by TCID50 assay in culture supernatants and cells homogenates, respectively. Overall, virus titers from supernatants and cells were reduced to a similar extent by GYY4137 treatment in a dose-dependent manner, independently of the virus strain tested (Fig. 1a). Specifically, we did not observe a significant reduction of virus replication of any strain tested at 1 mM concentration (data not shown), however 5 mM dose decreased H1N1, H3N2 and Brisbane virus titer in cell supernatants by about 10^2^ fold, and the 10 mM reduced H1N1 and Brisbane virus titer by up to 10^3^ fold, respectively, when compared to untreated cells. No significant difference in H3N2 virus titer was observed between the 5 and 10 mM dose. A similar result was observed for the cell-associated virus, where 5 and 10 mM treatment decreased H1N1, H3N2 and Brisbane virus replication between 10^2^ and 10^3^ fold, when compared to untreated cells.

As GYY4137 demonstrated a similar antiviral effect across different strains of influenza virus, subsequent experiments were performed using H1N1 and 10 mM dose. To investigate whether the antiviral activity was observed if the donor was administered several hours after infection, cells were treated at 3 and 6 h p.i. and harvested to measure viral replication. GYY4137 treatment still significantly reduced viral titers in both supernatants and cells when given at 3 h or 6 h p.i., compared to untreated cells (Fig. 1b), although with a lower efficiency at 6 h p.i. for the cell-associated virus. There was no statistically significant effect on viral replication when GYY4137 was given during adsorption only but not during infection (8.7 × 10^6^ vs 8.1 × 10^6^ for cells and 4.7 × 10^6^ vs 1.7 × 10^6^ for supernatant), indicating that GYY4137 does not affect viral entry.

**Figure 2:**
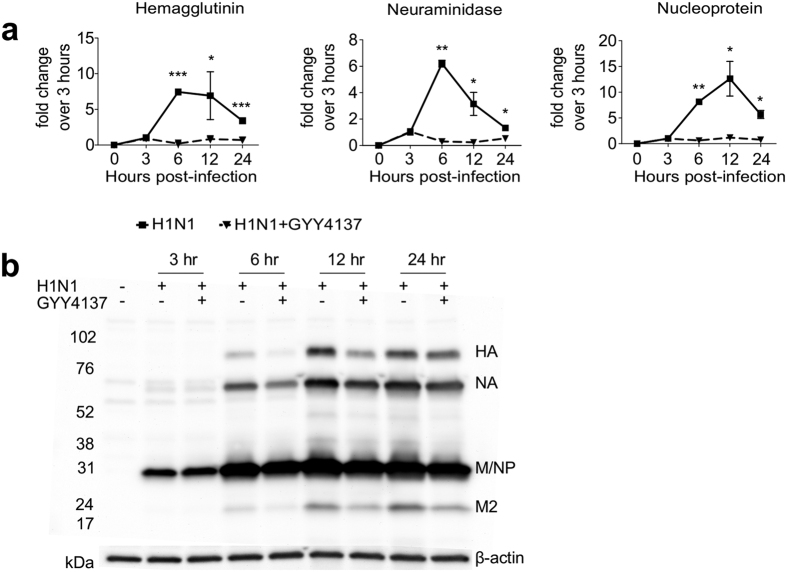
Effect of GYY4137 treatment on influenza viral RNA and protein expression. A549 cells were infected with influenza virus H1N1 at MOI of 1 and treated with 10 mM GYY4137 1 h p.i. Cells were harvested at the indicated time points after infection for total RNA and protein extraction. (**a**) Real-time PCR for H1N1 specific genes. Solid lines – H1N1 only, dashed lines – H1N1-infected cells treated with 10 mM GYY4137. The values represent fold change (2^−ΔΔ(Ct)^) of target viral RNA compared to the 3 h p.i. timepoint, after normalization using 18S RNA. Values represent arithmetic means, error – standard error of mean (SEM), n = 3; comparison are between H1N1-infected and GYY4137-treated samples at each time point *p < 0.05; **p < 0.01; ***p < 0.001 using separately for each time point. (**b**) Western Blot assay for H1N1 proteins. 15 μg of protein of cell lysates were used per lane. Membranes were probed using rabbit polyclonal anti-whole H1N1 antibody, and were stripped and reprobed with anti-human β-actin antibody for loading control.

To further investigate the effect of GYY4137 treatment on different steps of H1N1 viral replication, we assessed both viral mRNA and protein expression during a single cycle replication. A549 cells were infected with influenza H1N1 at high MOI and harvested at different time p.i. for RNA and total cell protein extraction. Using real-time PCR and H1N1 specific primers, we found a persistent reduction of hemagglutinin, neuraminidase and nucleoprotein mRNAs starting at 3 h p.i. and over 24 h of infection upon GYY4137 treatment (Fig. 2a), which correlated with a concomitant decrease in expression of the corresponding viral proteins, starting at 6 h p.i. (Fig. 2b).

### GYY4137 interferes with replication of other enveloped RNA viruses

**Figure 3:**
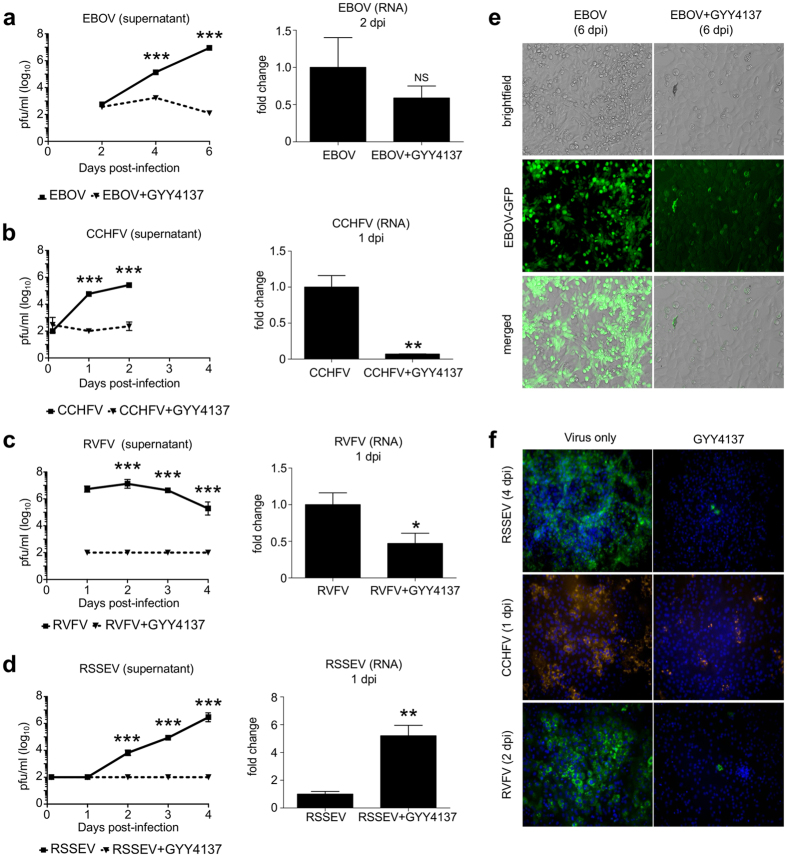
GYY4137 inhibits replication of highly pathogenic enveloped RNA viruses *in vitro*. (**a**–**d**) Viral titers and viral RNA expression of EBOV-eGFP, CCHFV 10200, RVFV ZH501 and RSSEV were assessed in Vero cells untreated or treated with GYY4137. Cells were infected with the above viruses at MOI of 1 and treated one hour later with 10 mM GYY4137 for EBOV or 5 mM for the other viruses. Cells and supernatants were collected at indicated time p.i. to extract RNA to assess viral RNA expression and to determine viral titers, respectively. Squares are virus-only samples, triangles are GYY4137-treated samples. For RNA expression data the values represent fold changes (2^−ΔΔ(Ct)^) of target RNA levels over control samples using delta-delta Ct method. 18S RNA was used as housekeeping control RNA. The values represent mean and SEM, n = 3; *p < 0.05; **p < 0.01; ***p < 0.001 treated versus untreated samples on the same day when compared using Mann-Whitney test. (**e**) Immunofluorescent and brightfield images of Vero cells infected with EBOV-GFP with or without GYY4137 at 6 day p.i. (**f**) Immunofluorescent staining of Vero cells infected with CCHFV, RVFV or RSSEV with or without GYY4137 using virus-specific antibodies at the indicated time points.

After demonstrating an antiviral effect of GYY4137 against influenza A and B, we evaluated the inhibitory effect of GYY4137 on viral replication against four highly pathogenic RNA viruses, namely EBOV (Recombinant Zaire Ebola virus), RSSEV (Far-eastern tick-borne flavivirus), CCHFV (Crimean-Congo hemorrhagic fever virus), and RVFV (Rift Valley fever virus). Vero cells were infected with the viruses at MOI of 1 and treated with either 10 mM (EBOV) or 5 mM (RSSEV, CCHFV and RVFV) of GYY4137 starting at 1 h p.i. Viral titers were assessed at up to 6 days p.i. by plaque assay. Similar to paramyxoviruses and influenza, GYY4137 treatment significantly reduced viral replication of all four viruses (Fig. 3a–d). Specifically, peak viral titers were reduced by 10^4^, 10^4^, 10^3^ and 10^4^ in GYY4137-treated EBOV-, RVFV-, CCHFV- or RSSEV-infected cells, compared to untreated. A striking reduction in viral replication for all viruses was also confirmed in cells at peak of viral replication by immunofluorescence assay (Fig. 3e,f). GYY4137 treatment significantly reduced levels of viral RNAs, determined by real-time PCR, during the earlier stages of RVFV and CCHFV replication, but it did not affect EBOV and RSSEV RNA levels (Fig. 3a–d), similar to what we observed in the course of RSV infection^14^.

### GYY4137 inhibits viral-induced pro-inflammatory mediator production

**Figure 4:**
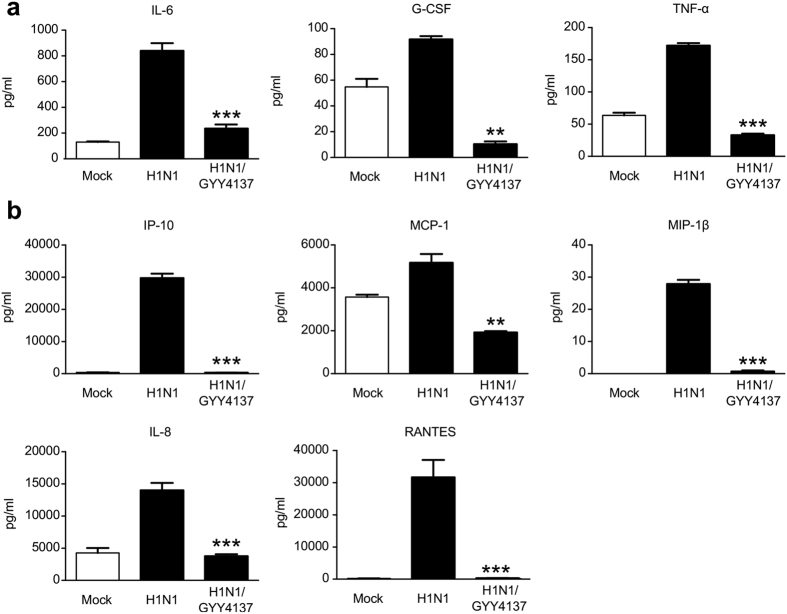
GYY4137 decreases influenza-induced cytokine and chemokine production. Confluent monolayers of A549 cells were infected with H1N1 at MOI of 1 and treated with 10 mM GYY4137 at 1 h p.i. The concentration of cytokines (**a**) and chemokines (**b**) was determined in cell supernatants collected 48 h later using Bio-Plex assay. Values represent arithmetic means, error – standard error of mean (SEM), n = 3; comparison between H1N1-infected and GYY4137-treated samples **p < 0.01; ***p < 0.001 treated versus untreated samples using one-way ANOVA with Tukey post-hoc analysis.

Upon entry into respiratory epithelial cells (upper and lower respiratory tract and alveolar cells), influenza triggers production of several pro-inflammatory cytokines and chemokines such as interferons, IL-1β, IL-8, IL-6 and TNF-α^19^. Cytokines and chemokines, in addition to danger- and pathogen-associated molecular patterns (DAMPs and PAMPS) released form virus-damaged cells, recruit and activate primary immune cells such as monocytes, neutrophils and dendritic cells, and act on neighboring endothelial cells causing them to express receptors that promote immune cell migration from circulation. Incoming neutrophils, monocyte-derived macrophages and cytotoxic T cells directly or indirectly damage respiratory epithelial and endothelial cells and further amplify the inflammatory response. Although inflammation is necessary for successful viral clearance, an excessive response is associated with damage of alveolar-endothelial barrier, causes accumulation of fibrin and fluid in the alveolar spaces with subsequent disruption of gas exchange and respiratory failure^19^. Therefore, inhibition of pro-inflammatory responses in influenza infection represents an important aspect of potential therapeutic interventions. To determine whether inhibition of viral replication was associated with a reduction in pro-inflammatory mediator secretion, A549 cells were infected with H1N1 influenza virus and treated with 10 mM GYY4137 at 1 h p.i. Cell supernatants were collected at 24 h p.i. and Bio-Plex multiplex assay was used to quantify a panel of cytokines and chemokines. GYY4137 treatment drastically reduced the secretion of the cytokines IL-6, TNF-α and G-CSF, as well as the chemokines IL-8, RANTES, IP-10, MCP-1 and MIP-1β from infected cells at 48 h p.i., compared to untreated cells (Fig. 4).

**Figure 5:**
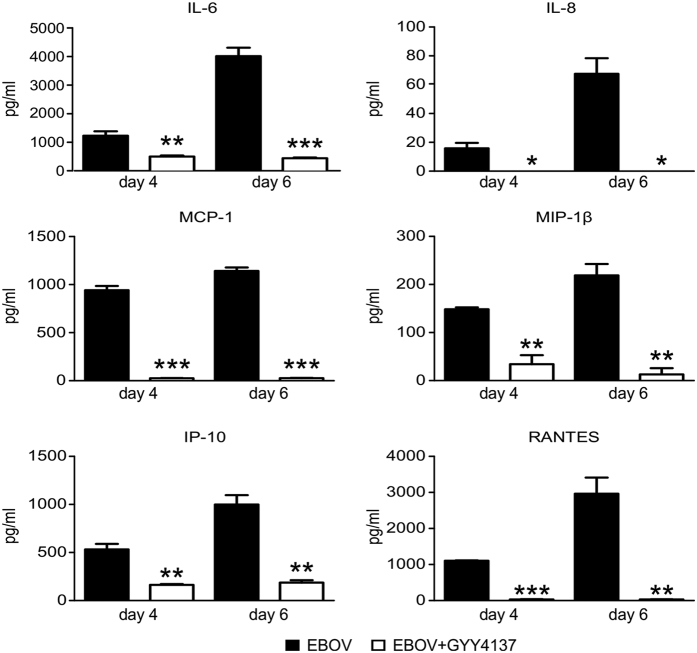
GYY4137 decreases EBOV-induced cytokine and chemokine production. Confluent monolayers of Vero cells were infected with EBOV and treated with 10 mM GYY4137 as described above. At 4 and 6 day p.i. the supernatant was collected and γ-irradiated. The concentration of cytokines and chemokines was determined using Bio-Plex system. Values represent arithmetic means, error bars – standard error of mean (SEM), n = 3; *p < 0.05; **p < 0.01; ***p < 0.001 treated versus untreated samples compared using one-way ANOVA with Tukey post-hoc analysis.

Inflammatory response plays detrimental role in the development of multi-organ failure and subsequent shock in EBOV infection. Upon infection of multiple cell types, including macrophages and dendritic cells, EBOV triggers the production of inflammatory mediators such as TNF-α, IL-6, IL-8 and IL-10^20^,^21^,^22^, while inhibiting host antiviral responses through several mechanisms including inhibition of interferon production and signaling, and apoptosis of immune cells (reviewed in ref. 23). This dysregulated and imbalanced immune response to the virus is a feature of fatal cases of EBOV infection by contributing to increased vascular leakage, disseminated intravascular coagulation, and shock^23^,^24^. To determine whether inhibition of viral replication was associated with a reduction in proinflammatory mediator secretion, Vero cells were infected with EBOV and treated with 10 mM of GYY4137. Cell supernatants were collected at 4 and 6 days p.i. and analyzed for cytokine and chemokine production. Indeed, GYY4137 treatment significantly reduced the secretion of IL-6, IL-8, RANTES, IP-10, MCP-1 and MIP-1β from infected cells compared to untreated, both at day 4 and 6 p.i. (Fig. 5).

### GYY4137 interferes with H1N1-induced cellular signaling

**Figure 6:**
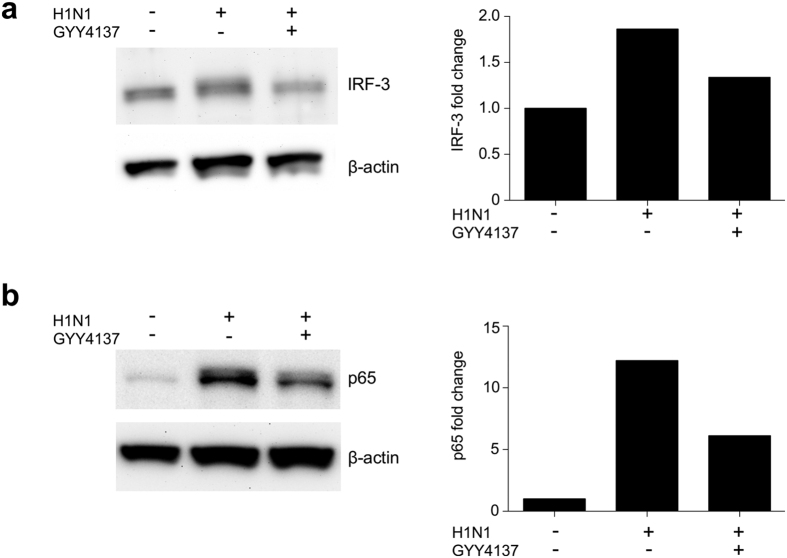
GYY4137 inhibits activation of influenza-induced signaling pathways. Confluent monolayers of A549 cells were infected with H1N1 at MOI of 1 and treated with 10 mM GYY4137 at 1 h p.i. Cells were collected at 48 h p.i. for nuclear protein extraction. Western Blot assays were performed using 25 μg of protein per lane of nuclear fraction. Membranes were probed either with rabbit polyclonal anti-IRF3 (**a**) or rabbit polyclonal anti-p65 antibody (**b**). Membranes were stripped and reprobed with anti-human β-actin antibody for loading control. The relative density of the bands for IRF-3 **(a)** and p65 **(b)** was calculated by normalizing to β-actin using ImageJ software. The images were cropped to show the bands of interest only. Full length blots are provided in Figs S1 and S2. Brightness and contrast were linearly adjusted for the whole image to enhance visibility.

The production of pro-inflammatory mediators during influenza infection of airway epithelial cells is tightly controlled by transcription factors belonging to the NF-κB and IRF families^25^,^26^. After virus endocytosis, production and release of viral RNA into cytoplasm triggers conformational changes in retinoic acid-inducible gene (RIG)-I^27^, which interacts with mitochondrial antiviral signaling protein (MAVS) and recruits tumor-necrosis factor receptor-associated factor 3 (TRAF-3) and later TBK-1 complex which causes phosphorylation of IRF-3. Recruitment of TRAF-6 activates NF-κB via IκB kinase complex. Double-stranded viral RNA is also sensed by transmembrane Toll-like receptors 3 (TLR3). DsRNA-TLR3 complex recruits adaptor molecule TIR-domain-containing adapter-inducing interferon-β (TRIF) which in turn undergoes oligomerization and recruits TBK1 or IKKɛ, and phosphorylates IRF-3. At the same time, recruitment of RIP1, TAK and IKK by TRIF adaptor molecule activates NF-κB^27^,^28^. Both, TLR-3-and RIG-1-dependent activation of IRF-3 and NF-κB triggers their translocation into the nucleus and binding to promoter regions genes encoding pro-inflammatory mediators such as cytokines and chemokines. To test whether GYY4137 treatment modulates activation of IRF-3 and NF-κB transcription factors in response to H1N1 infection, we prepared nuclear extracts from H1N1-infected A549 cells treated or not with GYY4137 and assessed their nuclear levels by western blot assay. A reduction in nuclear translocation level of both IRF-3 (Figs 6a and S1) and p65 (Figs 6b and S2), the major NF-κB transcriptional subunit, was observed in GYY4137-treated, compared to untreated infected cells, at 48 h p.i. These results suggest that the observed reduction of cytokines and chemokines after treatment with the H_2_S donor could be attributed to inhibition of NF-κB and IRF-3 transcription factor activation.

## Discussion

It is estimated that the annual influenza epidemics affects about 15% of the world population and contributes to substantial mortality in risk-groups with compromised immunity^29^. Currently polyvalent immunization is considered the most effective way of prevention of influenza infection and few antiviral treatment options are available^30^. However, influenza viruses are known for their high mutation rate which allows for the viral escape and the development of world-wide pandemics, such as the avian influenza A H5N1 and swine influenza A H1N1 viruses, to name a few^30^,^31^. A number of specific antiviral treatments utilizing M2 and NA inhibitors, targeting the immune system or combination of both are available^30^. However, treatments need to be administered early in the infection and certain strain of viruses developed a resistance to them. A recent Ebola outbreak in West Africa resulted in over 28,000 cases of the disease and more than 11,000 deaths^32^. Smaller sporadic outbreaks of Far-eastern subtype tick-borne flavivirus, RVFV, and CCHFV occur on a yearly basis and have previously been reported in Siberia/Russia, Africa/Arabian Peninsula, and Southeastern Europe, respectively^33^,^34^,^35^. The mortality rate can reach up to 20 and 50% for respectively RSSEV and CCHFV^36^,^37^, while RVFV rather causes a permanent debilitating disease in about 10% of cases^38^. Currently there is no specific antiviral treatment or vaccine for EBOV, as well as for other highly pathogenic viruses such as RSSEV, CCHFV, and RVFV^39^,^40^, therefore there is an urgent need for the development of broad-range safe and effective antiviral therapeutics.

In this study we tested the antiviral effect of the slow H_2_S-releasing compound GYY4137 on four families of RNA enveloped viruses. Recently, we demonstrated its effectiveness in paramyxovirus infections both *in vitro*^14^ and *in vivo*^15^. Exogenous administration of GYY4137 resulted in inhibition of both RSV replication and viral-induced production of pro-inflammatory cytokines and chemokines. Similarly, here we demonstrate that administration of GYY4137 inhibited replication of influenza A and B viruses and of the highly pathogenic viruses EBOV, RSSEV, CCHFV, and RVFV. In influenza virus infection, GYY4137 treatment reduced both released and cell-associated virus titers to fairly similar levels, suggesting that GYY4137 has an inhibitory effect on early stage of influenza infection. GYY4173 treatment post viral entry significantly reduced the amounts of influenza hemagglutinin, neuraminidase and nucleoprotein gene expression throughout the infection, which was paralleled by a reduction of the corresponding viral proteins, including M2. These results are different from what we observed during infection with RSV infection, whose treatment with GYY4137 resulted in significant inhibition of viral replication, which was not associated with a significant reduction in either viral genomic and mRNA or protein expression^14^. Reduced viral RNA expression after H_2_S donor treatment was also observed during CCHFV and RVFV infection. However there was no effect of GYY4137 administration on EBOV and RSSEV RNA levels, similar to RSV, suggesting multiple targets for H_2_S antiviral mechanism(s).

The striking reduction in influenza virus titers after H_2_S donor treatment suggests that other stages of replication are affected, such as assembly and release. M2 protein mediates nuclear transport of virus ribonuclear protein complexes^41^. M2 protein has also been shown to alter membrane curvature and assist in the budding of the virus^42^,^43^, therefore the observed reduction of M2 protein expression could lead to a diminished assembly and release of viral particles.

The robust inhibitory effect of GYY4137 on viral replication was paralleled by a significant decrease in the production of virus-induced pro-inflammatory mediators both in influenza and EBOV models of infection. In the context of a highly pathogenic influenza infections, severe illness has been correlated to a high viremia and a strong inflammatory response including high plasma levels of IL-1β, IL-6, IL-8, IP-10, TNF-α, MCP-1 and IFN-α^44^,^45^. Likewise, the severity of Ebola virus disease was statistically significantly associated with elevated plasma levels of several cytokines and chemokines such as IL-6, IL-8, IL-10, MCP-1 and MIP-1β during the acute phase of infection. These data suggest that GYY4137 treatment could have a significant impact on clinical illness associated with influenza and EBOV infections.

It has been previously shown that H_2_S and its donors exhibit anti-inflammatory activity both *in vitro* and *in vivo* in part by affecting cellular signaling responsible for expression of these mediators (reviewed in ref. 46). For example, GYY4137-associated inhibition of LPS-induced macrophage activation and bleomycin-induced pulmonary fibrosis was dependent on decreased NF-κB induction^47^,^48^. Transcription factors belonging to the IRF and NF-κB families play a significant role in the pathogenesis of influenza A virus infections by mounting an inflammatory response through TLR3 and RIG-I activation by viral RNA^19^,^27^, similar to other viruses including paramyxoviruses^49^,^50^,^51^,^52^. In this study, we found that GYY4137 treatment was associated with inhibition of influenza virus-induced NF-κB and IRF-3 nuclear translocation, likely reflecting the decreased levels of viral RNA, the major trigger of cellular signaling. These findings differ from what we observed in RSV infection, during which GYY4137 treatment was not associated with reduced viral RNA or NF-κB and IRF-3 nuclear translocation, although it significantly reduced NF-κB and IRF-3 binding to RANTES and IL- 8 endogenous promoters, leading to inhibition of gene expression^14^. The reduced binding was due to changes in post-translational modifications, such as inducible phosphorylation of serine residues, which in case of NF-κB modulates transcriptional activity without affecting its nuclear translocation^53^,^54^. H_2_S treatment is also associated with sulfhydration (or persulfidation), which appears to be an important post-translational modification modulating activity of cellular signaling proteins^55^,^56^. Future studies will address whether changes in transcription factor sulfhydration in the course of influenza and other virus infections represent an important mechanism of modulation of cellular signaling.

Although EBOV suppresses RIG-I signaling pathway at an early stage of infection^57^,^58^, it can induce pro-inflammatory mediator production by activating the TLR-4 pathway^59^,^60^, suggesting that the observed reduction of EBOV-induced cytokine and chemokine secretion could be due to inhibition of Toll-like receptor signaling.

In conclusion, in this study we found a robust antiviral activity of GYY4137 against four families of RNA enveloped viruses *in vitro*. Importantly, treatment with GYY4137 affected both viral replication as well as production of pro-inflammatory mediators and cellular signaling. Our results indicate that GYY4137 possess broad antiviral activity and should be explored as potential antiviral therapeutic approach in relevant preclinical models of viral infections.

## Methods

### Cells and viruses

MDCK, A549, Vero E6, Vero CCL-81, BHK and SW-13 cells were purchased from American Type Culture Collection (ATCC, Manassas, VA). Cells were grown in complete culture media (CCM) containing DMEM (Gibco/Thermo Fisher Scientific Waltham, MA) supplemented with Heat-Inactivated Fetal Bovine Serum (FBS) (Gibco) at 5% (MDCK) or 2% (Vero E6, Vero CCL-81, BHK and SW-13), 10 mM L-Glutamine (Gibco) and 100 IU/ml penicillin/streptomycin (Gibco). A549 cells were grown in F12K medium containing 10% heat-inactivated FBS, 10 mM L-glutamine, 100 IU/ml penicillin, and 100 μg/ml streptomycin.

Influenza H1N1 A/California/07/2009, H3N2 A/Victoria/361/2011 and B/Brisbane/60/2008 viruses were a generous gift from Dr. Pedro Piedra of Baylor College of Medicine, (Houston, TX). Viruses were grown in MDCK cells using modified CCM with 0% FBS, 2.5% BSA, 1 mM HEPES (Gibco) and 0.1% TPCK-treated trypsin from bovine pancreas (Sigma-Aldrich, St. Louis, MO). Recombinant Zaire Ebola virus expressing eGFP (EBOV-eGFP), and Rift Valley fever virus (strain ZH501; RVFV) were propagated in Vero E6 cells, Far-eastern tick-borne flavivirus (strain Sofjin; RSSEV) was propagated in BHK-S cells, and Crimean-Congo hemorrhagic fever virus (strain IbAr10200; CCHFV) was propagated in SW-13 cells. Virus titers were quantified by plaque assay using respectively Vero E6 (EBOV and RVFV), BHK (RSSEV) or SW-13 (CCHFV) cells, as previously described^61^,^62^,^63^. Titers were reported as log10 pfu/ml. All work with EBOV, RVFV, RSSEV and CCHFV was performed at biosafety level 4 (BSL4) facilities at UTMB.

### TCID50 assay

TCID50 method in MDCK cells was used to determine the titer of influenza viruses^64^. Serial 3-fold dilution of the sample was incubated with the cells in triplicate for seven days. The highest dilution of the virus that was still associated with 50% cytopathic effect in all three wells was used to estimate the amount of virus present in the original suspension according to Reed-Muench method^65^. To calculate the final titer of the virus the following formula was used: “TCID50/ml = 3^n^ × 1/V_in_” in which 3 – constant dilution factor, n – highest positive dilution of the virus determined on day 7 and V_in_ – initial amount of sample per replicate. Virus titers were expressed as median tissue culture infectious doses (log_10_ TCID50/ml).

### H_2_S donor treatment

GYY4137 [morpholin-4-ium-4-methoxyphenyl(morpholino)phosphinodithioate] was purchased from Sigma-Aldrich Inc. Stock solutions of 80 mM GYY4137 were prepared with sterile PBS (Gibco), passed through a 0.2 μM filter and further diluted with the appropriate cell culture media to a final concentration of 5 and 10 mM. We have previously tested the cytotoxicity of GYY4137 at the chosen concentrations using a LDH assay in A549 cells and no significant cytotoxic effect was observed^14^. For influenza virus infection, cells were treated with GYY4137 either during the adsorption time only, or starting at different time after adsorption, i.e. 1, 3 and 6 h post-infection (p.i.) and left in cell culture medium throughout the entire experiment. For all other viruses, treatment was initiated 1 h p.i. and continued throughout the duration of the experiment.

### Real-time (RT)-PCR assays

For EBOV, RVFV, CCHFV and RSSEV total cellular RNA from Vero CCL81 (ATCC) was extracted using TRIzol reagent (Invitrogen/Thermo Fisher Scientific), following the manufacturer’s recommendations. Quantitative reverse transcription (RT)-PCR assays were performed using a single TaqMan MGB probe and two unlabeled oligonucleotide primers (IDT, Coralville, IA) that recognize gene sequences corresponding to glycoprotein (GP) for EBOV, RNA-dependent RNA polymerase for RVFV, nucleocapsid protein for CCHFV, and the end of 3′ non-coding genomic segment for RSSEV, as described in refs 66, 67, 68 and 69, and as reported below:

EBOV-Fwd: TTTTCAATCCTCAACCGTAAGGC, Rev: CAGTCCGGTCCCAGAATGTG,EBOV-Probe: CATGTGCCGCCCCATCGCTGC,

RVFV-Fwd: TGARAATTCCTGARACACATGG, Rev: AYTTCCTTGCATCATCTGATG,RVFV-Probe: AATGTAAGGGGCCTGTGTGGACTTGTG,

CCHFV-Fwd: CAAGGGGTACCAAGAAAATGAAGAAGGC,Rev: GCCACAGGGATTGTTCCAAAGCAGAC,CCHFV-Probe: ATTTACATGCACCCTGCCGTGCTTACA,

RSSEV-Fwd: GGGCGGTTCTTGTTCTCC, Rev: ACACATCACCTCCTTGTCAGACT,RSSEV-Probe: TGAGCCACCATCACCCAGACACA.

EBOV, RVFV and CCHFV RNAs (both viral mRNA and viral genome) were quantified together using a One-step QuantiFast Probe RT-PCR kit (Qiagen, Hilden, Germany). RSSEV RNAs (viral genome and antigenome) were also quantified together using a Two-steps Quantifast Probe RT-PCR kit (Qiagen, Hilden, Germany) which included a viral RNA digestion step after cDNA synthesis. Quantification of 18S rRNA level was performed with the 18S rRNA gene TaqMan assay reagent (Thermo Fisher Scientific) and used as an endogenous control.

For influenza virus total RNA was isolated using Qiagen RNeasy Mini Kit (Qiagen) according to manufacturer’s instructions. After total RNA quantification using Nanodrop spectrophotometer (Thermo Fisher Scientific), 1 μg of total RNA was transcribed into cDNA using Invitrogen SuperScript III First-Strand Synthesis SuperMix (Thermo Fisher Scientific) and random hexamer primers, according to manufacturer’s instructions. Influenza gene amplification (from both viral mRNA and genome) was performed by RT-PCR using Power SYBR Green PCR Master Mix (Thermo Fisher Scientific) using the following primer sets obtained from Integrated DNA Technologies (IDT, Coralville, IA), as described in ref. 70:

hemagglutinin Fwd: GAGCTCAGTGTCATCATTTGAA, Rev: TGCTGAGCTTTGGGTATGAA; nucleocapsid Fwd: TGAAAGGAGTTGGAACAATAGCAA, Rev: GACCAGTGAGTACCCTTCCC; neuraminidase Fwd: CATGCAATCAAAGCGTCATT, Rev: ACGGAAACCACTGACTGTCC.

Duplicate threshold cycle (Ct) values were analyzed using the comparative delta-delta Ct method. The fold change of target gene expression was calculated using the 3 h p.i. time point as baseline, after normalization of values using the housekeeping 18S rRNA gene.

### Fluorescence Microscopy

Vero cells were seeded on cover slips, infected with viruses in BSL4 condition and fixed using 4% paraformaldehyde (PFA) for 48 h, and then for additional 24 h with fresh 4% PFA, before removal from the BSL4 laboratory and transfer to the BSL2 laboratory for immunostaining, mounting on slides and imaging on an Olympus IS71 microscope, as previously described^71^. Viral antigens were detected using either anti-RVFV NS mouse polyclonal antibody, anti-RSSEV Sophy mouse polyclonal antibody or anti-CCHFV rabbit polyclonal antibody (kindly provided by Drs. Tesh and Ksiazek, World Reference Center for Emerging Viruses and Arboviruses, UTMB), coupled to a secondary goat anti-mouse or anti-rabbit antibody (Thermo Fisher Scientific). Cells infected with EBOV-eGFP were directly imaged in the BSL4 laboratory. DAPI staining (Sigma-Aldrich) was added to localize cell nuclei in fixed samples.

### Western blot

Nuclear extracts of uninfected and infected cells were prepared by using hypotonic/nonionic detergent lysis, as previously described^72^,^73^. Total cell lysates were prepared from A549 cells by the addition of ice-cold RIPA buffer (Thermo Fisher Scientific), according to manufacturer’s instructions. After estimation of protein content with Bradford assay kit (Bio-Rad, Hercules, CA), 10 to 20 μg/sample were boiled in 2x Laemmli buffer and resolved on SDS-PAGE gels. Proteins were transferred onto a Hybond polyvinylidene difluoride membrane (Amersham, Piscataway, NJ), and nonspecific binding sites were blocked by immersing the membrane in Tris-buffered saline–Tween (TBST) containing 5% skim milk powder for 60 min. The membranes were then incubated with the primary antibody overnight at 4 °C, followed by incubation with the appropriate horseradish peroxidase (HRP)-conjugated secondary antibody (Sigma, St. Louis, MO) for 30 min at room temperature. After washing, proteins were detected using an enhanced chemiluminescence system (Amersham, GE Healthcare, United Kingdom) and visualized by UVP Scientia 600 Imaging System (UVP, Upland, CA). Primary antibodies used for Western blot assays were rabbit polyclonal anti-p65 (Cell Signaling Technology Inc.), rabbit anti-IRF-3 (Santa Cruz Biotechnology), rabbit polyclonal anti-H1N1 (Abcam), and mouse anti-β-actin (Sigma). The images were cropped to show the bands of interest only. Full length blots are provided in Figs S1 and S2. Brightness and contrast were linearly adjusted for the whole image to enhance visibility. Image J software was used for image adjustment and to analyze protein band density^74^.

### Bio-Plex assay

Cytokine and chemokine concentrations in cell-free supernatants were determined using the Bio-Plex Cytokine Human 8-plex immunoassay (Bio-Rad), according to the manufacturer’s instructions. The concentration of 8 analytes (IL-6, IL-8, TNF-α, IP-10, MCP-1, MIP-1β, G-CSF and RANTES) were quantified. Samples from EBOV-infected cells were inactivated on dry ice by gamma irradiation (5 Mrad) prior to removal from the BSL4 laboratory for analysis at BSL2 approved facilities.

### Statistical Analysis

Unpaired parametrical (t-test) or non-parametrical (Mann-Whitney) tests were used to compare two groups at a time. One-way analysis of variance (ANOVA) with Tukey’s post-hoc analysis was used in multiple comparisons. Null hypotheses were rejected at *P* values less than 0.05. All data are presented in figures represent means ± standard error (*p < 0.05, **p < 0.01, ***p < 0.001). Influenza experiments were performed a minimum of three times, while those performed under BSL4 conditions were repeated two to three times. Statistical analysis was performed with GraphPad Prism 5 software (GraphPad Software, Inc., La Jolla, CA). Curve fitting was performed using Microsoft Excel Software. Images and charts were combined and annotated using NIH Image J^74^.

## Additional Information

**How to cite this article:** Bazhanov, N. *et al*. Broad-Range Antiviral Activity of Hydrogen Sulfide Against Highly Pathogenic RNA Viruses. *Sci. Rep.* **7**, 41029; doi: 10.1038/srep41029 (2017).

**Publisher's note:** Springer Nature remains neutral with regard to jurisdictional claims in published maps and institutional affiliations.

## Supplementary Material

Supplementary Material
