# Supplementary material for: Broad-Range Antiviral Activity of Hydrogen Sulfide Against Highly Pathogenic RNA Viruses
Source: Sci Rep. 2017 Jan 20;7:41029. doi: 10.1038/srep41029 (PMC5247713; doi:10.1038/srep41029)
Supplement: Supplementary Material [file srep41029-s1.pdf]

Broad-Range Antiviral Activity of Hydrogen Sulfide Against Highly Pathogenic RNA Viruses

Nikolay Bazhanov[1a], Olivier Escaffre[2a], Alexander N. Freiberg[2], Roberto P. Garofalo[1,3,4] and Antonella Casola[1,3,4*]

[1]Department of Pediatrics, [2] Department of Pathology, [3] Microbiology and Immunology and [4]Sealy Center for Vaccine Development, University of Texas Medical Branch, Galveston, TX

[a] These authors have equally contributed to the work.

*Correspondence should be addressed to Antonella Casola, M.D., Department of Pediatrics, University of Texas Medical Branch, 301 University Blvd., Galveston, TX 77555. E-mail: ancasola@utmb.edu

Supplementary figure 1. GYY4137 inhibits activation of IRF-3.

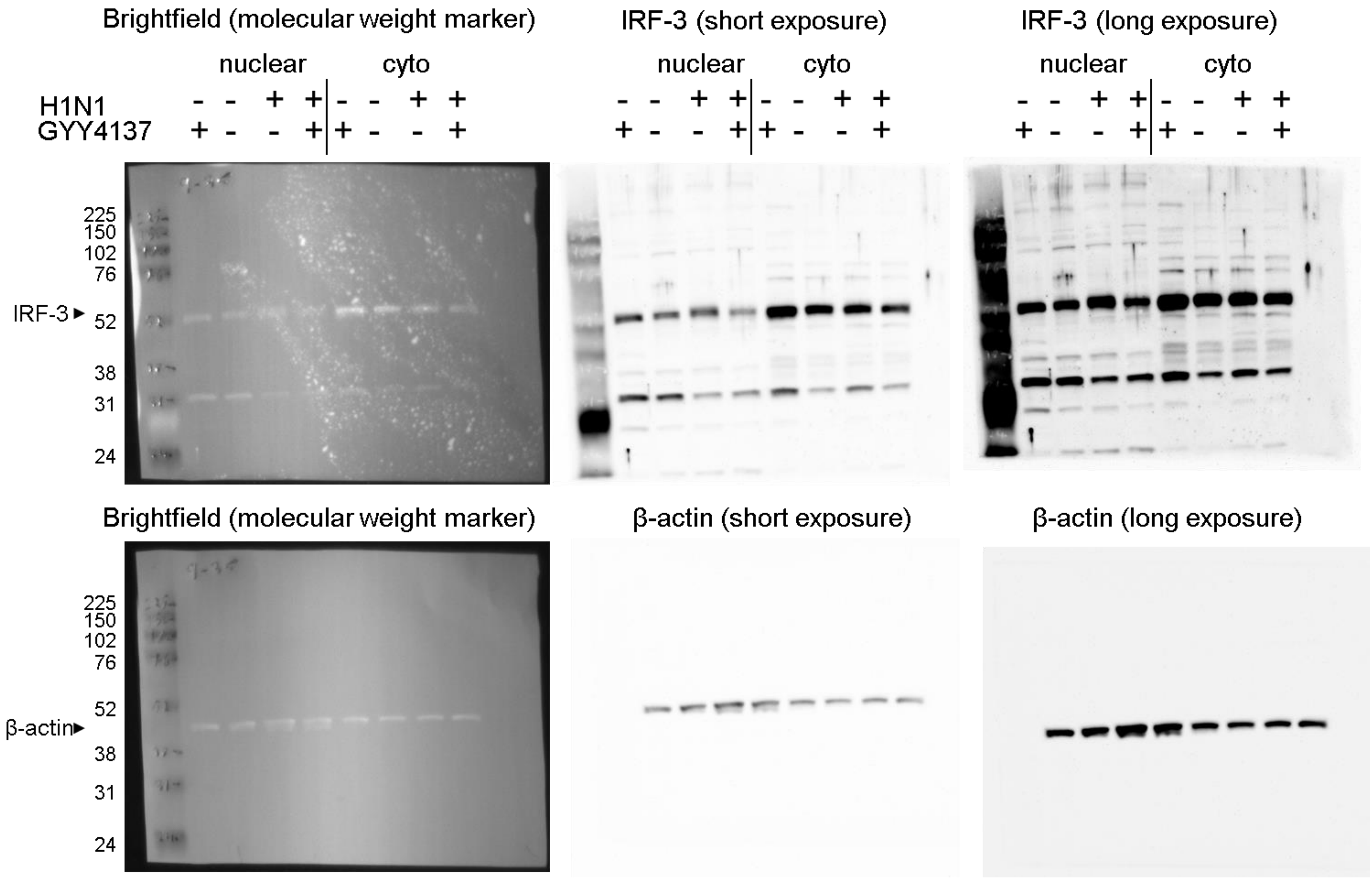

Supplementary figure 1. GYY4137 inhibits activation of IRF-3. Confluent monolayers of A549 cells were infected with H1N1 at MOI of 1 and treated with 10 mM GYY4137 at 1h p.i. Cells were collected at 48h p.i. for nuclear and cytoplasmic protein extraction. Western Blot assays were performed using 25 µg of protein per lane of nuclear fraction. Membranes were probed with rabbit polyclonal anti-IRF3 antibody. Membranes were stripped and reprobed with anti-human β-actin antibody for loading control. Full lengths of the membranes corresponding to the cropped out bands on **Fig 6a** are shown.

Supplementary figure 2. GYY4137 inhibits activation of NF-κB.

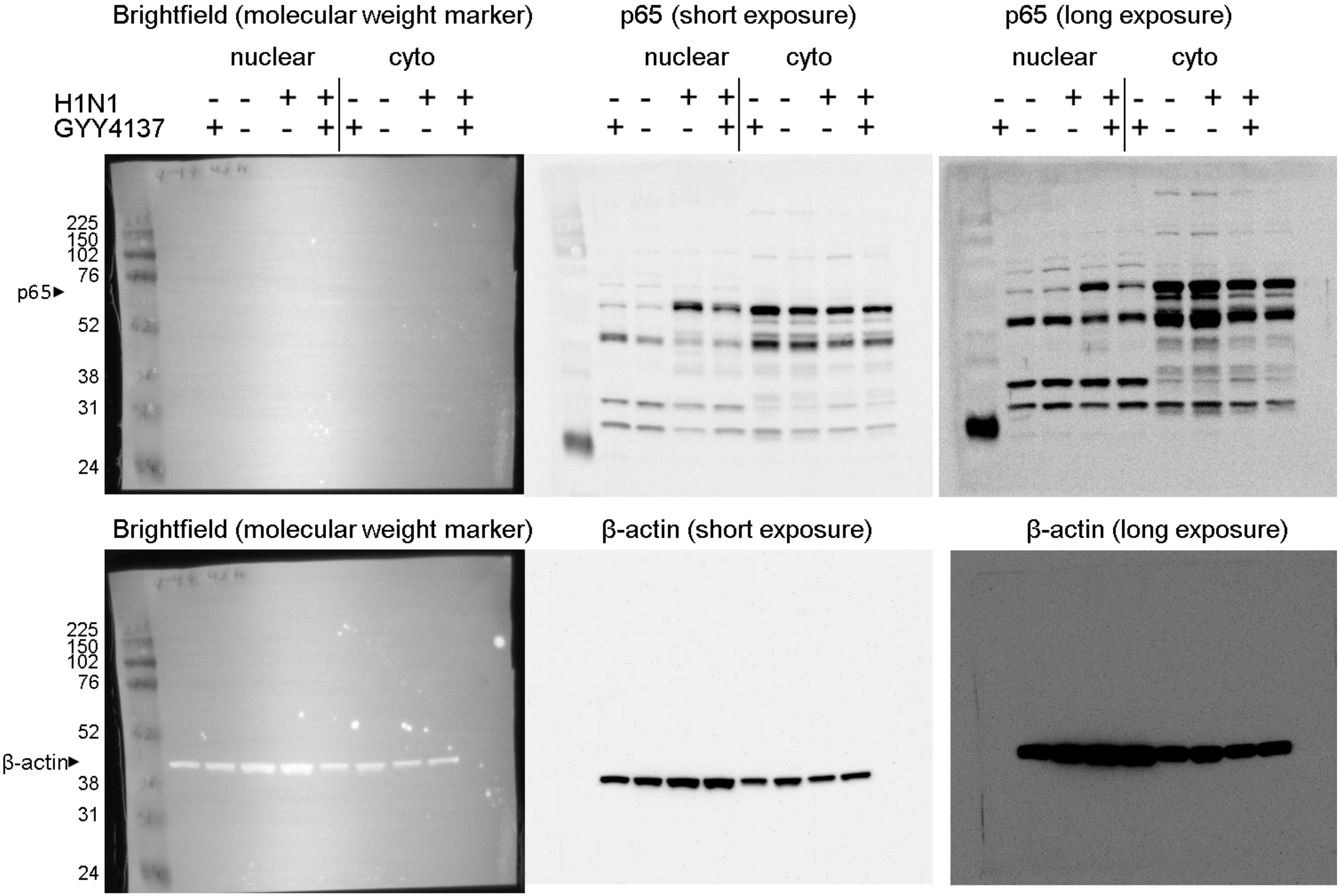

Supplementary figure 2. GYY4137 inhibits activation of NF-κB. Confluent monolayers of A549 cells were infected with H1N1 at MOI of 1 and treated with 10 mM GYY4137 at 1h p.i. Cells were collected at 48h p.i. for nuclear and cytoplasmic protein extraction. Western Blot assays were performed using 25 µg of protein per lane of nuclear fraction. Membranes were probed with rabbit polyclonal anti-p65 antibody. Membranes were stripped and reprobed with anti-human β-actin antibody for loading control. Full lengths of the membranes corresponding to the cropped out bands on **Fig 6b** are shown
